# Assessing of the Italian version of the Memory Strategy Test (TMS) in people with Parkinson disease: a preliminary descriptive psychometric study

**DOI:** 10.1007/s10072-023-06906-6

**Published:** 2023-06-24

**Authors:** Maria Grazia Vaccaro, Luca Pullano, Silvia Canino, Massimiliano Pastore, Alessia Sarica, Andrea Quattrone, Sara Margarida Fernandes, Filippo Migliorini, Fernando Maestu, Aldo Quattrone

**Affiliations:** 1 Neuroscience Research Centre, Department of Medical and Surgical Sciences, Magna Graecia University, Viale Europa, Germaneto, Catanzaro 88100 Italy; 2 Department of Health Sciences, Magna Graecia University of Catanzaro, Catanzaro, 88100 Italy; 3 Department of Developmental and Social Psychology, Padova University, Padua, Italy; 4 Portucalense University; INPP – Portucalense Human Development Institute, Porto, Portugal; 5 Department of Orthopaedic, Trauma, and Reconstructive Surgery, RWTH University Hospital, 52074 Aachen, Germany; 6 Department of Experimental Psychology, Faculty of Psychology, Complutense University of Madrid, Madrid, Spain; 7 Center for Cognitive and Computational Neuroscience, Complutense University of Madrid, Madrid, Spain

**Keywords:** Memory Strategies Test, Parkinson’s disease, Neuropsychological Assessment, Executive Functions, Psychometric Properties

## Abstract

**Background:**

Previous literature has shown that executive functions (EF) are related to performance in memory (M) tasks. The Test of Memory strategies (TMS) is a psychometric test that examines EF and M simultaneously and it was recently validated on an Italian healthy cohort. The first aim of the study was to apply TMS, for the first time, on a sample of patients with Parkinson's disease (PD), who are characterized by mild cognitive impairment. The second aim is to investigate whether TMS scores can discriminate PD patients from healthy controls.

**Method:**

Ninety-eight subjects were enrolled, including 68 patients with PD, and 30 Italian healthy controls (HC), who also underwent a memory evaluation through well-known tests**.**

**Results:**

Confirmatory factor analysis (CFA) demonstrated that TMS of PD patients had a bi-dimensional structure as previously found in healthy cohort. In detail, The TMS-1 and TMS-2 lists require greater involvement of the EF factor, while TMS-3, TMS-4 and TMS-5 the M factor. Receiver operating characteristic (ROC) curves and precision-recall (PR) curves showed that the M subscale can distinguish between HC and PD, while EF had poor discrimination power.

**Conclusion:**

The hypothesized prediction model of TMS test seems to have adequate ability to discriminate PD from HC especially for the M function.

**Supplementary Information:**

The online version contains supplementary material available at 10.1007/s10072-023-06906-6.

Recently, a new psychological test that evaluates memory (M) and executive functions (EF) simultaneously was developed by Yubero and colleagues [1] called Test of Memory Strategies (TMS). The development of the TMS assumes that the greater the damage to the EF, the worse the subject's ability to use internal storage strategy [2, 3]. Furthermore, the ability to generate cognitive strategies for encoding information in memory and retrieving it depends on the executive system, which allows us to optimize the resolution of novel tasks.

The study conducted by Yubero [1] highlights how EF influences the performance of memory tasks in elderly subjects with different neurological profiles. The research group of Fernandes [3] developed a Portuguese version of the TMS to evaluate the effect of aging in a population of healthy subjects. The Portuguese version of the TMS appears to be a suitable tool for simultaneously assessing memory and executive functions, both in physiological and pathological aging. The Italian version of the TMS was also developed and has been applied on a sample of 121 healthy subjects aged between 18 and 89 years [4]. The factor analysis confirmed the presence of a bi-dimensional model (EF and M) with excellent fit indices.

The TMS in Italian version has so far been applied only on a sample of healthy subjects, there are no studies with application on Parkinson's disease (PD) is the most common neurodegenerative movement disorder [5]. The disease usually onset between the ages of 50 and 60 and has a chronic and progressive evolution. Parkinson’s disease is characterized by tremor at rest, rigidity, and bradykinesia and cognitive deficits [6]. Visual-spatial, memory and executive function deficits are among the most important and have a major impact on the subject's quality of life and ability in normal activities of daily living [7–11]. Historically, clinicians have used several types of tests to evaluate the global cognitive function, such as Mini Mental State Examination (MMSE) which although a screening tool, is able to assess PD cognitive impairment [12–14]. However, other tests are needed, for example to thoroughly investigate the memory [15–17], the ability to develop strategies and executive functions, etc. [17–20]. Given these premises, TMS would be a suitable test to simultaneously evaluate executive functions and verbal memory of pathological samples.

In clinical contests usually the patient must make several different visits at the same time (resonance imaging, electromyography, neurological visit, etc.), thus TMS appears to be a useful tool also in temporal and economic terms.

For all these reasons, the main aims of the present study are to apply TMS on PD patients for the first time, to describe their psychometric characteristics through TMS taking as reference the study by Vaccaro and colleagues [4], and finally to assess the discrimination power of TMS in distinguishing PD from healthy controls (HC).

## Materials and methods

### Participants

The study included 102 Italian participants (36 females) with mean age of 65.4 (SD = 8.47 Min = 47, Max = 86). The Patients were enrolled from the Movement Disorders Unit of University, between January 2021 to June 2022 and were classified in 68 with idiopathic PD (22 females; mean age = 65.8, SD = 8.87, Min = 47, Max = 86), and 34 age/level of education-matched healthy control subjects (HC, 14 females; mean age = 64.7, SD = 7.67, Min = 51, Max = 84).

Clinical diagnoses for PD patients were established according to international diagnostic criteria (Postuma et al., 2015). All patients met criteria for PD with magnetic resonance (MR) support at the time of evaluation. Exclusion criteria were the presence of other neurological, psychiatric, or comorbid disorders and brain injury. We have used the MMSE useful for global cognitive screening as already demonstrated in the literature [14]. For healthy participants, individuals with a score of the Mini Mental State Examination 2 (MMSE-II) [21] lower than 24/30, affected by neurological or psychiatric diseases, taking medications in recent years were excluded. All participants read and signed the written informed consent. The study was approved by the Regional Research Ethic Committee in accordance with the criteria set laid down in the 1964 Declaration of Helsinki.

### Instruments

All participants were evaluated by the same neuropsychologist with more than 12 years of experience in assessment of neurological and neurodegenerative disease. All patients were evaluated by the neurologist with more than 20 years of experience in movement disorders. All participants underwent the MMSE, standardized cognitive screening neuropsychological test, and a TMS.

### Test of Memory Strategies

The TMS test (in Supplementary Materials—SM) is a tool developed to evaluate the impact of EF and M on cognitive performance, to measure whether a deficit found in a memory task can be attributed to a primary memory problem or to a secondary EF deficit, and vice versa. As already described [1, 3, 4] the TMS consists of five lists of words, presented in series which must be listened by the participant; each single list is made up of 10 different and randomly distributed words: (a) TMS-1: an incidental learning task consisting of 10 words without any semantic and/or phonetic relation between them. The participants are not aware that they are performing a memory task, but they think that execution is a linguistic task. This condition provides information about learning in the absence of explicit executive strategies. (b) TMS-2: an explicit learning task in which the 10 words in the list have no semantic and/or phonetic relation between them. The participants know they are performing a memory task. There is a need for an internal organization of memory strategies in this condition, the involvement of memory and EF is required. (c) TMS-3: a task with 10 words belonging to two semantic categories – trees and interior decoration. The words are presented randomly, and words in each category are mixed. Participants are not instructed to say that there are distinct semantic categories. There is a reduction of the need for memory strategies in this condition, as the list of words is organized into two different semantic categories. In TMS-2 and TMS-3, there is a higher need for working memory. (d) TMS-4: in this task, the words are organized into two semantic categories, but unlike TMS-3, words are not presented randomly. The first five words consist of the transport category, while the remaining five words consist of the category of tools. Participants are not instructed that there are two different semantic categories. There is a reduction in memory strategies because the material is externally organized in two consecutive semantic categories. (e) TMS-5: in the latter list, the words are organized like in TMS-4 and presented in two categories in a structured way, the first five words belong to the category of sports, while the remaining five belong to vegetables. In this condition, the psychologist makes the participant aware that there are two distinct semantic categories without knowing that the categories are sports and vegetables. In this final condition, as in TMS-4, there is a lesser need for internal cognitive strategies due to the external organization of the material. The TMS-5 is the condition that most minimizes the need for executive functioning.

The Italian version of TMS has been validated and applied on the Italian healthy population already by Vaccaro and colleagues [4]. The evaluation was conducted in a single session lasting about 60 min.

### Statistical analyses

The statistical analyses were performed with R software (v. 2022.07.1.554 for Macintosh) [22] and JAMOVI software (version 1.6.15,2020). Descriptive analyses for the whole sample, for each study group (PD, HC), considering age, gender and education level were calculated, means, skewness, kurtosis, and minimum and maximum score obtained for each TMS list by participants.

T-tests were employed for comparing age and education levels among groups. Differences in the gender distribution between groups were assessed with pairwise Pearson Chi-square (p < 0.05).

In line with the previous study conducted by Vaccaro et al. [4] a set of Pearson Correlations between the different scores obtained in each single list of TMS (TMS List 1, TMS List 2, TMS List 3, TMS List 4, TMS List 5) were calculated to determine the relationship between word lists.

Furthermore, we conducted a preliminary confirmatory factor analysis (CFA) performed using maximum likelihood (MLR) estimator to evaluate the dimensional structure (EF and M) of TMS found in the previous study [4] and applied to a group of patients with Parkinson’s disease.

Goodness of fit indices was assessed through, Tucker–Lewis index (TLI), comparative fit index (CFI), standardized root mean square residual (SRMR) and root mean square error of approximation (RMSEA), as well as the model acceptability evaluated through the following cutoff criteria: TLI > 0.95, CFI > 90, SRMR < 0.08; RMSEA < 0.08. Indices of CFA obtained in the study of Vaccaro et al. [4] have been taken as reference for our CFAs results.

We hypothesized different linear models with the aim to investigate the role of pathology, gender, age and education level on the EF and M subscales scores. The Akaike Information Criterion (AIC) was used to select the best-fit model for our data.

Finally, the receiver operating characteristics (ROC) curves were used to evaluate the discriminative capacities of the two subscales of TMS (EF and M) to understand their diagnostic power among HC and PD patients. Specifically, the optimal thresholds of ROC curves, and consequently the specificity (from 0 to 1 is good index) and sensitivity (from 0 to 1 is good index), has been calculated according to the Yuden Index.

However, due to the unbalanced sample, we also used the Precision-Recall Curves (PR; from 0 to 1 is a good index) which are adequate in cases like this. In fact, according to Saito and Rehmsmeier [23] the PRs are more explicitly informative than ROCs when the classes, or group considered, are unbalanced overcoming this issue. In the choice of classes both for ROC and PR, we considered a negative class the HC group.

## Results

### Descriptive statistics

The distribution of genders did not show significant differences between M e F [χ2; *p* = 0.51], just as age (p = 0.40) and education level (p = 0.53) did not report significant differences.

**Fig. 1:**
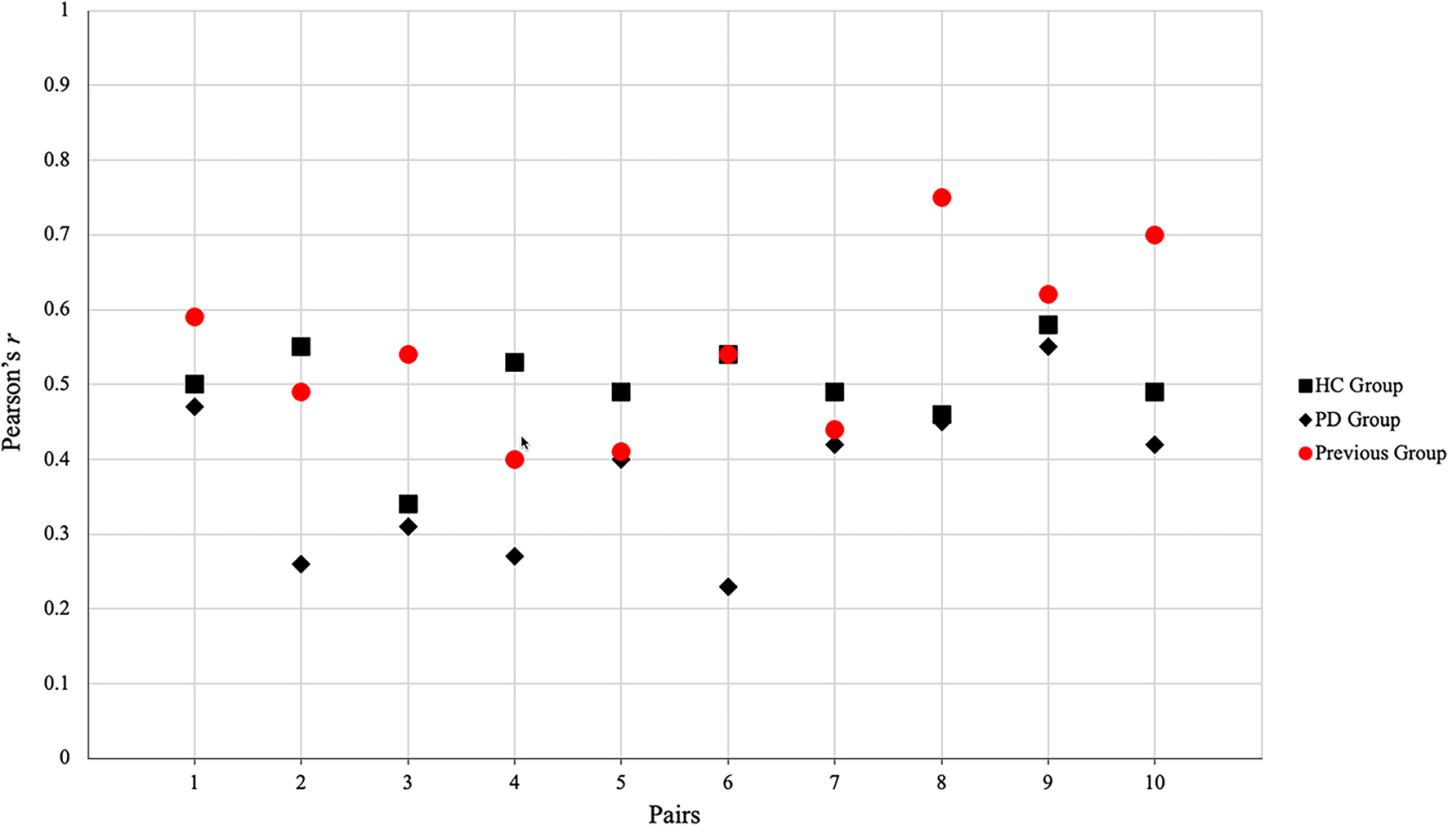
Pearson correlations between TMS lists for HC, PD group and previous sample [4]. Pair 1: TMS list 1 – TMS list 2; Pair 2: TMS list 1 – TMS list 3; Pair 3: TMS list 1 – TMS list 4; Pair 4: TMS list 1 – TMS list 5; Pair 5: TMS list 2 – TMS list 3; Pair 6: TMS list 2 – TMS list 4; Pair 7: TMS list 2 – TMS list 5; Pair 8: TMS list 3 – TMS list 4; Pair 9: TMS list 3 – TMS list 5; Pair 10: TMS list 4 – TMS list 5

The descriptive analysis about lists of TMS test (Table 1) showed how the mean number of words repeated by subjects increased from TMS-1 to TMS-4 and how it decreased from TMS-4 to TMS-5, as depicted in Fig. 1SM. Particularly, the TMS scores obtained from participants of this study, are compared with TMS scores from healthy participants of the study of Vaccaro et al. [4]. It is worth noting that the healthy subjects from the previous study were younger than the healthy cohort here used.

**Table 1:** Descriptive Statistics of demographic and clinical data, MMSE and TMS lists of HC group (n = 34) and PD group (n = 68)

	Participants	Mean	Median	SD	Skewness	SE-skew	Kurtosis	SE-kurt	Min	Max
Age	HC	64.7	64	7.67	0.36	0.40	0.22	0.79	51	84
	PD	65.8	66.5	8.88	-0.15	0.29	-0.57	0.57	47	86
Education Level	HC	11.8	13	3.67	-0.72	0.40	-0.32	0.79	5	17
	PD	11.1	13	4.02	-0.22	0.29	-1.02	0.57	3	17
MMSE	HC	28.1	28.5	1.96	-0.64	0.40	-0.73	0.79	24	30
	PD	24.2	25	4.33	-1.67	0.29	3.92	0.57	7	39
	PD	3.75	3.5	2.72	0.59	0.29	-0.13	0.57	0	11
TMS-1	HC	3.32	3	1.12	-0.01	0.40	-0.91	0.79	1	5
	PD	2.50	2	1.57	0.52	0.29	0.13	0.57	0	7
TMS-2	HC	3.79	4	1.25	0.71	0.40	0.15	0.79	2	7
	PD	3.13	3	1.18	-0.15	0.29	-0.36	0.57	0	5
TMS-3	HC	4.56	5	1.08	0.06	0.40	0.67	0.79	2	7
	PD	4.07	4	1.08	0.21	0.29	0.12	0.57	2	7
TMS-4	HC	5.94	6	1.23	0.01	0.40	-0.03	0.79	3	8
	PD	4.68	5	1.62	-0.59	0.29	-0.13	0.57	0	7
TMS-5	HC	5.56	6	1.52	-0.50	0.40	-0.26	0.79	2	8
	PD	4.09	4	1.37	0.23	0.29	-0.69	0.57	2	7
TMS-T	HC	23.2	23	4.81	-0.15	0.40	0.34	0.79	12	32
	PD	18.5	18.5	4.82	0.21	0.29	-0.50	0.57	9	30
EF	HC	7.12	7	2.06	0.41	0.40	0.18	0.79	3	12
	PD	5.63	5	2.37	0.42	0.29	0.22	0.57	0	12
M	HC	16.1	16	3.15	-0.58	0.40	0.64	0.79	8	21
	PD	12.8	13	3.28	-0.06	0.29	-0.69	0.57	5	19

*Abbreviations: MMSE* = Mini-Mental State Examination Raw Score-II; *TMS-1* = total sum of words from the first list remembered; *TMS-2* = total sum of words from the second list remembered; *TMS-3* = total sum of words from the third list remembered; *TMS-4* = total sum of words from the fourth list remembered; *TMS-5* = total sum of words from the fifth list remembered; *TMS-T* = TMS Total sum of words remembered of all lists; *EF* = Sum of the score of the TMS 1 and 2; *M* = Sum of the score of the TMS lists 3,4 and 5; *SD* = Standard Deviation; *SE-skew* = Skewness error standard; *SE-kurt* = Kurtosis error standard; Min = Minimum score; Max = Maximum score

### Correlation between TMS lists

We conducted the Pearson Correlations Analyses considering all TMS lists in each group (HC, PD). In Fig. 1 are reported our correlations results compared with the Pearson correlations obtained by Vaccaro and colleagues [4] on healthy sample.

### Confirmatory Factor Analyses (CFA)

As regards the structure of the TMS, the preliminary CFAs were performed on the PD group.

The CFA highlighted the bi-dimensional structure, namely the factor 1 in reference to EF (TMS-1 and TMS-2 lists) and factor 2, in reference to M (TMS-3, TMS-4 and TMS-5). Furthermore, the CFAs for PD group showed excellent fit indices (TLI: 1; CFI: 1; RMSEA: 0; SRMR: 0.027) comparable to the previous CFA (TLI: 1; CFI: 1; RMSEA: 0; SRMR: 0.017) conducted on the healthy Italian Sample [4]. In the following table (Table 2) are reported the factor loadings of the bi-dimensional model obtained in the present study on PD group and in the previous study of Vaccaro and colleagues’ study [4].

**Table 2:** Factor Loadings of CFAs of our and previous study

Factor	Indicator	Estimate	SE	*Z*	*p*	Stand. estimate
Present study: PD group (n = 68)						
EF	TMS List 1	0.917	0.222	4.14	< .001	0.589
	TMS List 2	0.938	0.185	5.06	< .001	0.798
M	TMS List 3	0.806	0.134	6.02	< .001	0.749
	TMS List 4	0.927	0.204	4.54	< .001	0.578
	TMS List 5	1.007	0.169	5.94	< .001	0.741
Previous study [4]: n = 121						
EF	TMS List 1	1.1	0.127	8.69	< .001	0.780
	TMS List 2	1.14	0.135	8.45	< .001	0.759
M	TMS List 3	1.56	0.153	10.19	< .001	0.802
	TMS List 4	2.04	0.160	12.74	< .001	0.933
	TMS List 5	1.50	0.159	9.40	< .001	0.756

*Abbreviations: EF* = Executive Functions, the sum of the score of the TMS lists 1 and 2; *M* = Memory, the sum of the score of the TMS lists 3,4 and 5; *TMS-1* = total sum of words from the first list remembered; *TMS-2* = total sum of words from the second list remembered; *TMS-3* = total sum of words from the third list remembered; *TMS-4* = total sum of words from the fourth list remembered; *TMS-5* = total sum of words from the fifth list remembered

### Prediction model

To evaluate the factors that could predict EF and M scores of our study, we performed separate linear models for each subscale on the whole sample (n = 102), to investigate the main effects with different combinations of the independent variables: presence of pathology, gender, age, and education level. The best-fit model was selected using AIC index both for EF and M subscales. All models hypothesized are reported in the table below for EF and M (Table 3).

**Table 3:** Hypothesized general linear models for EF and M subscale

Hypothesized Model	Subscale	AIC	Subscale	AIC
Null	EF	468.26	M	541.75
PT	EF	460.93	M	533.25
Gender	EF	468.00	M	552.89
Education Level	EF	463.93	M	536.16
Age	EF	456.34	M	545.48
PT + Gender	EF	461.30	M	534.93
PT + Education level	EF	457.34	M	533.26
PT + Age	EF	449.15	M	526.85*
Gender + Education level	EF	464.61	M	552.64
Gender + Age	EF	456.64	M	546.95
Education level + Age	EF	455.40	M	546.59
PT + Gender + Age	EF	449.99	M	528.79
PT + Gender + Education level	EF	458.45	M	548.31
PT + Education level + Age	EF	448.71*	M	528.42
Gender + Age + Education level	EF	456.27	M	530.84
PT + Gender + Age + Education level	EF	450.00	M	530.50

^*^Best model

*Abbreviations*: *AIC* = Akaike Information Criterion; *EF* = Executive Functions, the sum of the score of the TMS lists 1 and 2; *M* = Memory, the sum of the score of the TMS lists 3,4 and 5; *PT* = Pathology; *Null* = A model without independent variables

The best-fit model of EF subscale, with an AIC index of 448.09 (*R*^*2*^ = 0.22, *F*_[3,98]_ = 9.504, *p* < 0.001), included the main effects of the presence or absence of pathology (PD [B = -1.33, *t* = -2.99, *p* < 0.003]); education level (B = 0.01, *t* = 1.7, *p* = 0.04); and age (B = -0.085, *t* = -3.315, *p* = 0.001).

On the other hand, the best-fit model of M subscale, with an AIC index of 526.86 (*R*^*2*^ = 0.25, *F*_[2, 99]_ = 16.42, *p* < 0.001), included the main effects of the presence or absence of pathology (PD [B = -3–10, *t* = -2.944, *p* < 0.001]); and age (B = -0.11, *t* = -2.94, *p* = 0.004).

## Results of ROC and PR curves

### EF Subscale

**Fig. 2:**
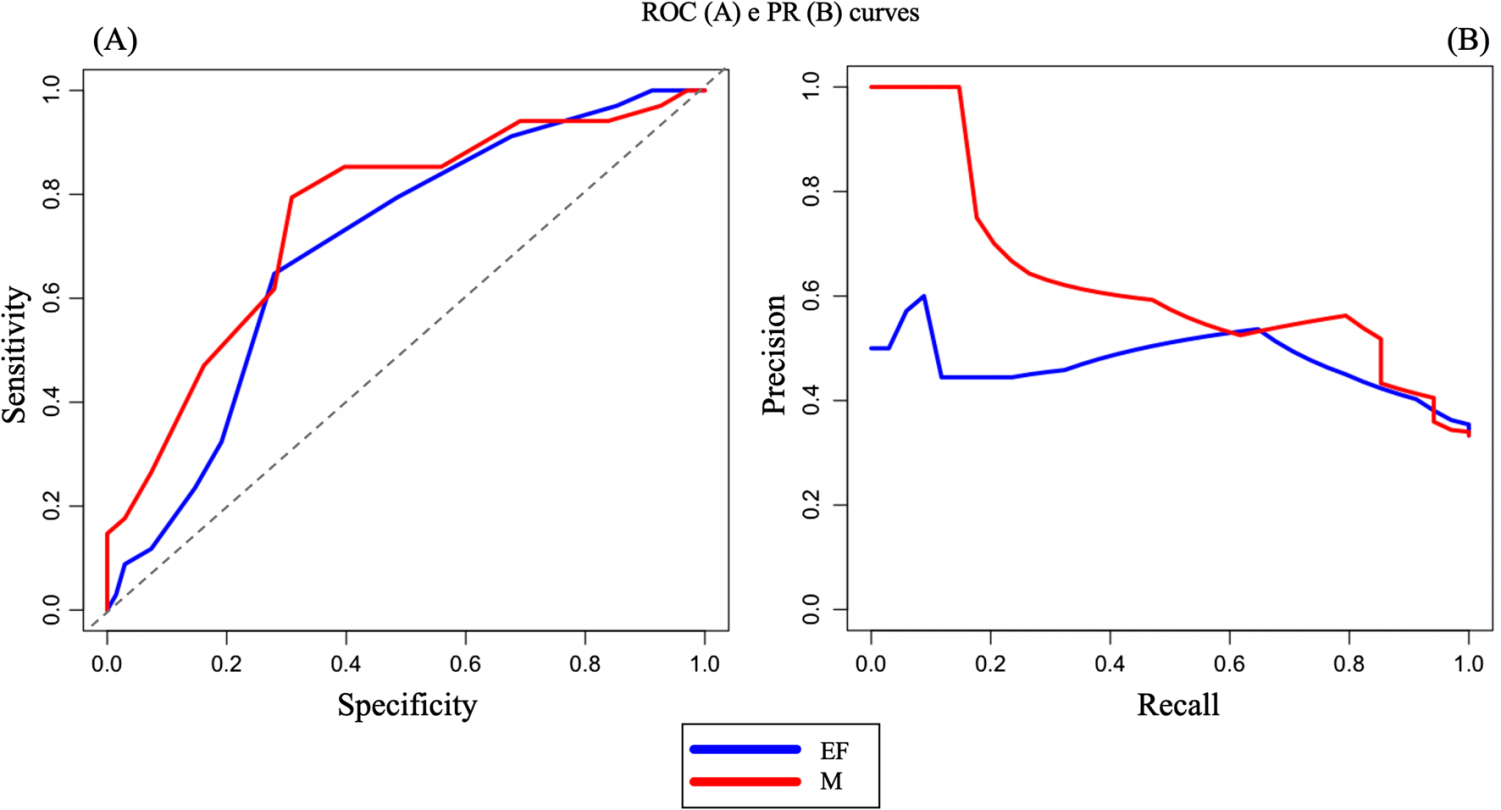
The ROC curves of EF subscale (panel **A**) and M subscale (panel **A**), and the PR curves of EF subscale (panel **B**), M subscale (panel **B**)

ROC curves reported that EF subscale seemed able to discriminate between HC group (negative class) and PD group (positive class) by area under curve (AUC) equaling 0.70 (Fig. 2, panel A, blue line). The Youden Index method suggested an optimal threshold (0.66) based on specificity of 0.65 and sensitivity of 0.72.

On the other hand, PR curves reported different results. The PR conducted on EF among HC group (negative class) and PD group (positive class) reported a Precision-Recall area under the curve (PRAUC) of 0.47 (Fig. 2, panel B, blue line), with a precision of 0.42 and a recall of 0.82. Specifically, the thresholds and F1-scores of PRs are reported in Table 4, together with the parameters of ROC.

### M Subscale

ROC curves reported that M subscale seemed able to discriminate between HC group (negative class) and PD group (positive class) by area under curve (AUC) equaling 0.76 (Fig. 3, panel A, green line). The Youden Index method suggested an optimal threshold (0.67) based on specificity of 0.79 and sensitivity of 0.70. Also, in this case the PR curves reported different compared to the ROCs. The PR conducted on M among HC group (negative class) group and PD group (positive class) reported a Precision-Recall area under the curve (PRAUC) of 0.62 (Fig. 2, panel B, green line), with a precision of 0.54 and a recall of 0.82. The thresholds and F1-scores of PRs are reported in Table 4.

**Table 4:** ROC and PR curves indices of EF and M subscales between HC and PD groups

ROC Curve	AUC	Threshold	Specificity	Sensitivity	
EF	0.70	0.66	0.65	0.72	
M	0.76	0.67	0.79	0.70	
PR Curve	PRAUC	Threshold score	Precision	Recall	F_1_
EF	0.47	5	0.42	0.82	0.57
M	0.62	14	0.54	0.82	0.65

*Abbreviations*: *EF* = Executive Functions, the sum of the score of the TMS lists 1 and 2; *M* = Memory, the sum of the score of the TMS lists 3,4 and 5; AUC = Area under the curve, *PRAUC* = Precision-Recall area under the curve; *F*_1_ = F_1_ score, a measure of accuracy

## Discussion

As we have already seen from the study of Sarica et al. (2021), the MMSE is considered an adequate cognitive screening tool in the neurological field [14], but more specific tests are needed to know more about memory and executive functions and how they both interact. In our study, we investigated the psychometric properties of TMS applied on PD patients and the clinical utility of TMS in discriminating PD from healthy participants. In detail, we observed the two main functions M and EF measured by TMS.

We followed in the footsteps of Yubero and colleagues who applied and analyzed TMS on a Spanish sample. Data were analyzed using an interpreted descriptively, rather than inferentially, given the exploratory intent of the study [24].

As already evident in the literature, also in our study the group of healthy subjects showed higher scores on the MMSE screening test [10, 14]. As for the TMS, PD obtained progressively high scores on the TMS lists from 1 to 4 while they decayed from 4 to 5 as previously studied [1, 3, 4]. The result also in this case shows the same characteristics of the previous study of Vaccaro and colleagues. We hypothesized that the cause is in the instructions given to the subjects in the fifth word list of the TMS (TMS-5) in which they were asked to do several things at the same time. Furthermore, it could be due to the “focusing effect” as subjects show excessive attention to a minimum of detail rather than considering instructions in general. For example, instead of focusing attention on the list of words to remember, study participants may have focused their attention on the category in which to place the words and thus not memorizing them. Indeed, future studies will aim to modify the instructions given for the last word list and compare two groups of subjects to test our hypothesis.

In the study by Vaccaro et al. [4] the CFA was performed on a group of healthy subjects, while our goal, in this study, was to make a preliminary verification of the goodness of the CFA indices on a PD sample From the confirmatory analysis in our study, we obtain excellent fit indices comparable to those on a healthy sample obtained by Vaccaro et al. [4]. In detail, the results of the CFA in the PD groups confirm the bi-dimensional structure of the head of the memory strategies already investigated by Vaccaro et al. [4]. In line with Yubero et al. [1] and Fernandes et al. [3], our results support the idea of the TMS as a measure of memory and executive functions also useful in Parkinson’s disease. Among the hypothesized predictive models, the best ones (models with lowest AIC) showed that the presence or absence of the disease, age and education level seem to influence the scores obtained by subjects on the EF subscale. For example, younger participants without disease, or with disease, and with a high level of education had higher scores on the EF subscale. On the other hand, the best-fit model for M-scores highlighted the contribution of the presence or absence of pathology and an effect of age. In this case younger participants without disease, or with disease, scored higher on the M subscale. It is interesting to note that the level of education seems to play a role on the score obtained on the EF subscale, i.e., on the ability to organize and plan necessary to mentally order the words in the TMS lists, but does not, however, seem to influence M, on the other hand, seems to be influenced by the presence of the disease and by age.

Receiver Operating Characteristics (ROC) curves and Precision-Recall (PR) curves were used to evaluate the ability of EF and M subscale, the total score of TMS, and the score of RAVLT I to discriminate between healthy controls (HC) and pathological groups. An approach with PR curves seems to be more appropriate precisely because the clinical sample is small and unbalanced, i.e., Parkinson’s patients are more than double the healthy controls. The two types of analysis conducted, have reported different results. In fact, as above cited, PR curves are more explicit when the classes, or group, are unbalanced, and they are used as more accurate statistics for our study. According to the ROC curve, the EF subscale seemed able to discriminate between HC and PD group, but not for the PR curve that reported lower index, except for the recall (the *true positive rate* identified), although the threshold score was very low (5 points on a total of 20). On the other hand, the M subscale seemed appropriate to discriminate between HC and PD according to the ROC curve and for the PR curve although with a high rate of *false positives* and a good threshold score of 14 points on a total of 30.

Instead, the total score of TMS (the sum of EF and M) to be able to discriminate HC group from PD group according to ROC curve with and PR curve although also in this case there is a high rate of *false positive* and a good threshold score of 26 points of on a total of 50.

Globally, the analyses conducted to understand the discriminative power of TMS subscale EF and M in different groups (healthy subjects and pathological ones) were in contrast, although the recommended PR curves are more informative for study like ours. The M subscale seemed more discriminative among the groups than EF subscale, suggesting how memory is easier to investigate for a neuropsychological test than executive functions that may require tasks more complex. Indeed, the total score of TMS seems to discriminate better between PD and HC than between EF and M.

However, despite the PR curve being recommended for unbalanced samples, further studies with a larger sample and balanced sample are required to confirm or contradict the preliminary results obtained in our study.

Some important limitations need to be highlighted. The numerical sample is small. Although our main goal was to compare healthy subjects with a clinical group. The control group is not as numerous as the group of Parkinson's patients because it is very difficult to enroll completely healthy elderly subjects, with a low level of education and available to come to the research center.

The next future aim is to increase the sample of healthy subjects and find a cut-off that helps us to define within which range the score obtained at the TMS can be considered healthy or deficient in the EF and M functions. In detail, if the sum of TMS -1; -2 and TMS-3; -4; -5 reflects to EF and M respectively, a specific future goal is to define a cut-off for TMS-1; -2 (EF) and TMS -3; -4; -5 (M) separately. Identifying a specific cut-off for each area is useful to understand when there is a greater involvement of EF or M in the execution of the task.

Neuropsychological tests are inexpensive, non-invasive, and more easily administered compared to other methods like PET or RMN. To differentiate between neurodegenerative disorders with similar cognitive profiles via neuropsychological testing would allow clinicians to act at a lower cost and in less time.

In conclusion, the TMS seems to be a useful test to investigate the involvement of memory and executive functions simultaneously even in subjects with neurodegenerative diseases. The main reason for the TMS is to elucidate whether the memory deficits is caused by a primary memory problem or by an EF dysfunction and vs. Our results alongside a refinement of the instrument's scoring could help explain whether cognitive impairment in Parkinson's is more related to a deficit in primary memory or executive functions. A single test could be useful also to reduce the number of tests to be administered to the patient in the assessment phase and TMS may have a direct relevance to clinical practice. For example, at our research center patients come from multiple Regions near and far and in a single day the patients are subjected to more instrumental examinations, so having a shorter neuropsychological battery could be very useful for reducing evaluation times and waiting for caregivers.

### Supplementary Information

Below is the link to the electronic supplementary material.

Table S1 (DOCX 17 kb)

Fig. S1 (PNG 698 kb)

High Resolution (TIF 79 kb)
