# Supplementary material for: Assessing of the Italian version of the Memory Strategy Test (TMS) in people with Parkinson disease: a preliminary descriptive psychometric study
Source: Neurol Sci. 2023 Jun 24;44(11):3895–903. doi: 10.1007/s10072-023-06906-6 (PMC10570218; doi:10.1007/s10072-023-06906-6)
Supplement: Supplementary file 1 — (DOCX 17 kb) [file 10072_2023_6906_MOESM1_ESM.docx]

| 1. Blocco 2. Paese 3. Chimica 4. Polemica 5. Errore 6. Capriccio 7. Momento 8. Pubblico 9. Colpa 10. Stella   1.  2.  3.  4.  5.  6.  7.  8.  9.  10. | 1. Essenza 2. Senso 3. Consiglio 4. Grado 5. Forma 6. Esempio 7. Impurità 8. Fede 9. Tonnellata 10. Prezzo   1.  2.  3.  4.  5.  6.  7.  8.  9.  10. | 1. **Quercia** 2. Poltrona 3. Letto 4. **Noce** 5. Attaccapanni 6. **Pesco** 7. **Salice** 8. Tavolo 9. **Pino** 10. Armadio   1.  2.  3.  4.  5.  6.  7.  8.  9.  10. | 1. **Automobile** 2. **Treno** 3. **Autobus** 4. **Elicottero** 5. **Tram** 6. Martello 7. Tenaglie 8. Seghetto 9. Cacciavite 10. Pinza   1.  2.  3.  4.  5.  6.  7.  8.  9.  10. | 1. **Atletica** 2. **Calcio** 3. **Nuoto** 4. **Rugby** 5. **Pallamano** 6. Cavolo 7. Sedano 8. Fagioli 9. Carciofi 10. Melanzana   1.  2.  3.  4.  5.  6.  7.  8.  9.  10. |
| --- | --- | --- | --- | --- |
| Totale:  Intrusioni: | Totale:  Intrusioni: | Totale:  Totale Categoria 1:  Totale Categoria 2:  Intrusioni: | Total:  Total Categoria 1:  Total Categoria 2:  Intrusioni: | Total:  Total Categoria 1:  Total Categoria 2:  Intrusioni: |

Table 1SM Italian version of the Test of the Memory Strategies
