# Supplementary figures and images for: Assessing of the Italian version of the Memory Strategy Test (TMS) in people with Parkinson disease: a preliminary descriptive psychometric study
Source: Neurol Sci. 2023 Jun 24;44(11):3895–903. doi: 10.1007/s10072-023-06906-6 (PMC10570218; doi:10.1007/s10072-023-06906-6)

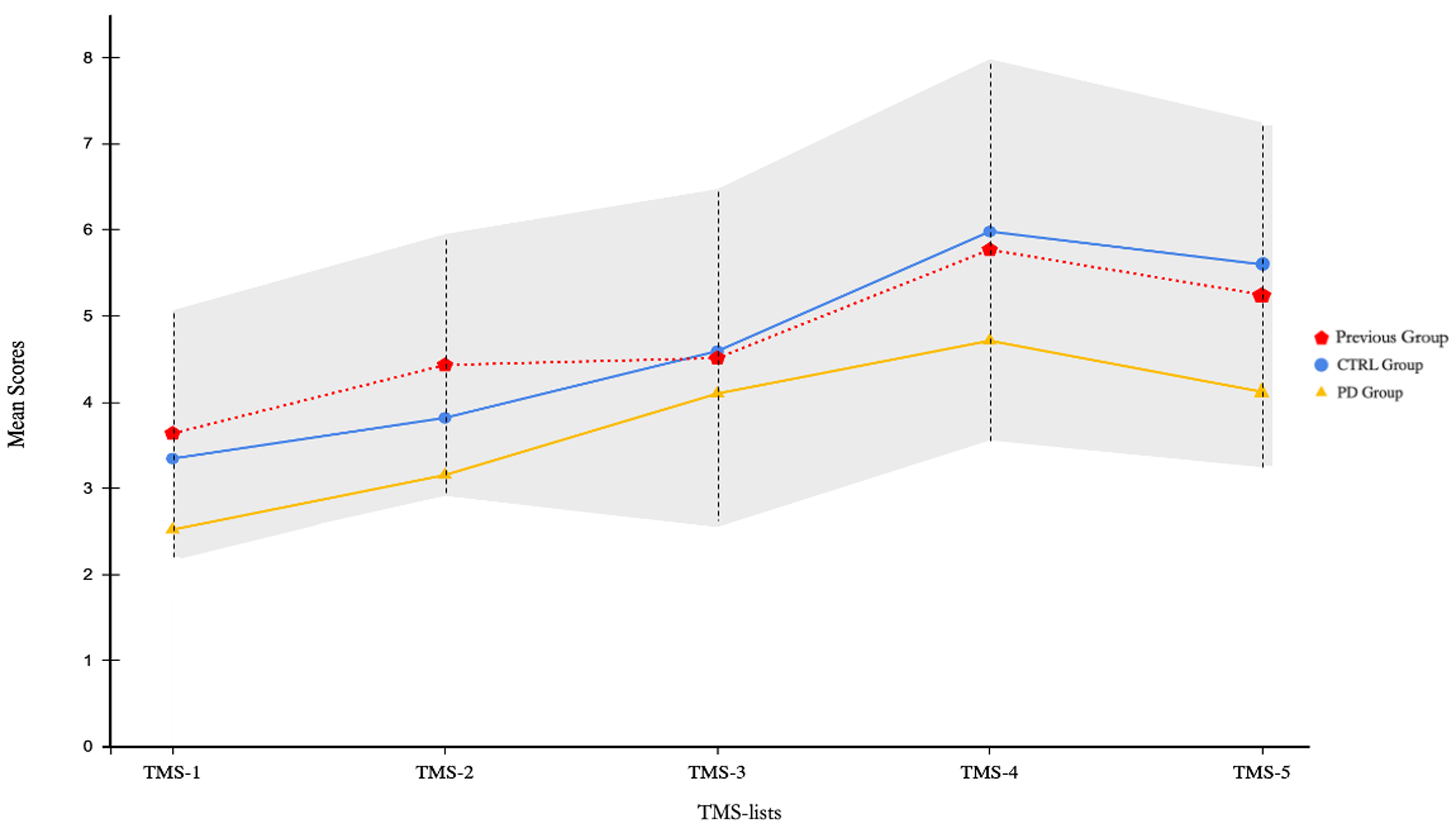

Supplement: Supplementary file 2 — (PNG 698 kb) [file 10072_2023_6906_Fig3_ESM.png]

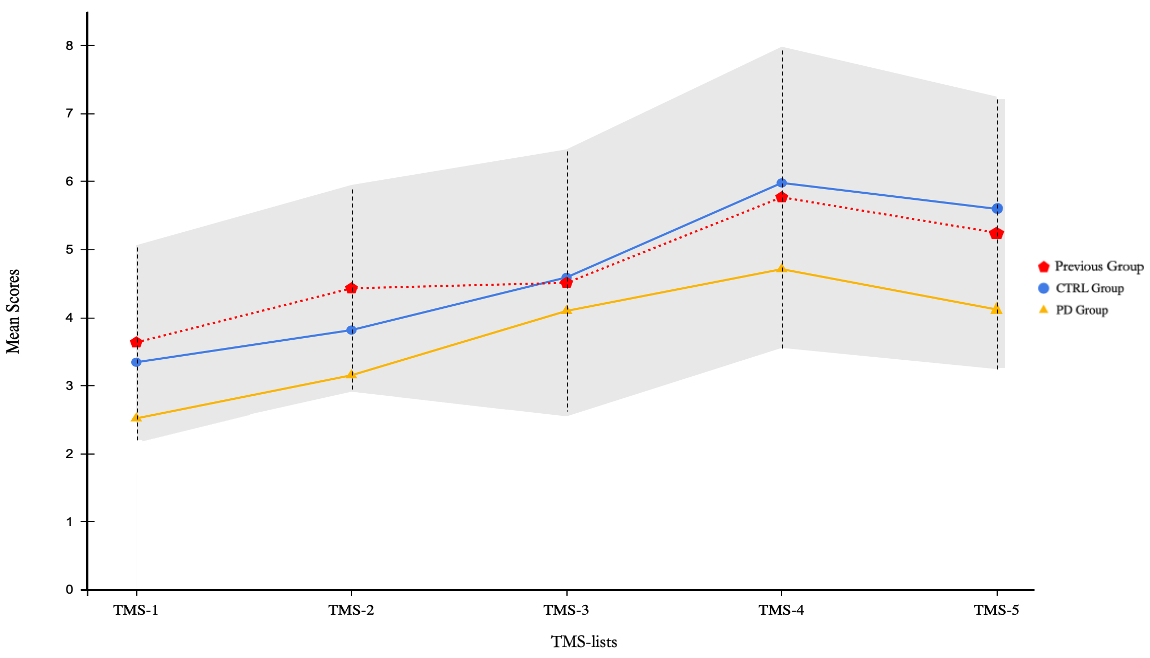

Supplement: Supplementary file 3 — High Resolution (TIF 79 kb) [file 10072_2023_6906_MOESM2_ESM.tif]
